# Audiovisual biofeedback breathing guidance for lung cancer patients receiving radiotherapy: a multi-institutional phase II randomised clinical trial

**DOI:** 10.1186/s12885-015-1483-7

**Published:** 2015-07-18

**Authors:** Sean Pollock, Ricky O’Brien, Kuldeep Makhija, Fiona Hegi-Johnson, Jane Ludbrook, Angela Rezo, Regina Tse, Thomas Eade, Roland Yeghiaian-Alvandi, Val Gebski, Paul J Keall

**Affiliations:** 1 Radiation Physics Laboratory, Sydney Medical School, The University of Sydney, Sydney, NSW Australia; 2 Central Coast Cancer Centre, Gosford Hospital, Gosford, NSW Australia; 3 Department of Radiation Oncology, Calvary Mater Newcastle, Newcastle, NSW Australia; 4 Department of Radiation Oncology, Canberra Hospital, Canberra, ACT Australia; 5 Department of Radiation Oncology, Chris O’Brien Lifehouse, Sydney, NSW Australia; 6 Department of Radiation Oncology, Northern Sydney Cancer Centre, Sydney, NSW Australia; 7 Radiation Oncology Network, Crown Princess Mary Cancer Centre, Westmead Hospital, Sydney, NSW Australia; 8 Department of Radiation Oncology, Nepean Cancer Care Centre, Sydney, NSW Australia; 9 University of Sydney NHMRC Clinical Trials Centre, Sydney, NSW Australia

**Keywords:** Breathing guidance, Motion management, Randomised, Stratified, Phase II clinical trial, Lung cancer, Radiotherapy

## Abstract

**Background:**

There is a clear link between irregular breathing and errors in medical imaging and radiation treatment. The audiovisual biofeedback system is an advanced form of respiratory guidance that has previously demonstrated to facilitate regular patient breathing. The clinical benefits of audiovisual biofeedback will be investigated in an upcoming multi-institutional, randomised, and stratified clinical trial recruiting a total of 75 lung cancer patients undergoing radiation therapy.

**Methods/Design:**

To comprehensively perform a clinical evaluation of the audiovisual biofeedback system, a multi-institutional study will be performed. Our methodological framework will be based on the widely used Technology Acceptance Model, which gives qualitative scales for two specific variables, perceived usefulness and perceived ease of use, which are fundamental determinants for user acceptance. A total of 75 lung cancer patients will be recruited across seven radiation oncology departments across Australia. Patients will be randomised in a 2:1 ratio, with 2/3 of the patients being recruited into the intervention arm and 1/3 in the control arm. 2:1 randomisation is appropriate as within the interventional arm there is a screening procedure where only patients whose breathing is more regular with audiovisual biofeedback will continue to use this system for their imaging and treatment procedures. Patients within the intervention arm whose free breathing is more regular than audiovisual biofeedback in the screen procedure will remain in the intervention arm of the study but their imaging and treatment procedures will be performed without audiovisual biofeedback. Patients will also be stratified by treating institution and for treatment intent (palliative vs. radical) to ensure similar balance in the arms across the sites. Patients and hospital staff operating the audiovisual biofeedback system will complete questionnaires to assess their experience with audiovisual biofeedback. The objectives of this clinical trial is to assess the impact of audiovisual biofeedback on breathing motion, the patient experience and clinical confidence in the system, clinical workflow, treatment margins, and toxicity outcomes.

**Discussion:**

This clinical trial marks an important milestone in breathing guidance studies as it will be the first randomised, controlled trial providing the most comprehensive evaluation of the clinical impact of breathing guidance on cancer radiation therapy to date. This study is powered to determine the impact of AV biofeedback on breathing regularity and medical image quality. Objectives such as determining the indications and contra-indications for the use of AV biofeedback, evaluation of patient experience, radiation toxicity occurrence and severity, and clinician confidence will shed light on the design of future phase III clinical trials.

**Trial registration:**

This trial has been registered with the Australian New Zealand Clinical Trials Registry (ANZCTR), its trial ID is ACTRN12613001177741.

## Background

The precision of radiotherapy can be reduced due to respiratory-related tumour motion, particularly for tumours in the thoracic region, leading to increased irradiation of healthy surrounding tissues, resulting in a significant increase in radiation-related toxicity [1–3]. This is further exacerbated when respiration is irregular in nature (deep/shallow breaths, baseline shifts, suspended breathing, etc.) [4, 5]. A 1Gy increase in tumour dose results in a 4 % improvement in survival, [6] however, a 0.5 cm range of tumour motion can cause a 4 ~ 5 % variation in radiation dose [7] which leads to an increase in mean dose to healthy surrounding tissues resulting in an increase in risk of pneumonitis and radiation toxicity [8, 9].

Techniques such as respiratory gating, breath-holds and tumour tracking are clinically useful for tumour motion management [10, 4, 11]. However, irregular respiration can reduce the efficiency of such motion management techniques, [12, 13] irregular respiration also causes motion artefacts and anatomic errors in medical imaging [14–19].

**Fig. 1:**
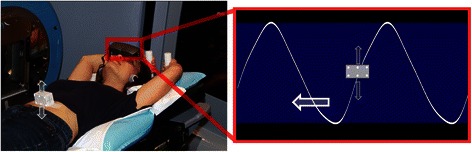
AV biofeedback system (left). Display goggles and real-time position management (RPM) marker block on the abdomen shown. The visual display (right), as seen by the patient, of the AV biofeedback guiding interface shows the waveguide (white curve) and a marker position (grey marker) in real time

Breathing guidance is one such technique which specifically aims to produce regular patient breathing by showing the patient how to adjust their breathing in real-time. One such breathing guidance system is the audiovisual (AV) biofeedback system (shown in Fig. 1), developed by Venkat, et al [13].

AV biofeedback is a real-time, interactive and personalised respiratory guide designed to facilitate regular patient breathing. Table 1 outlines the findings from previous AV biofeedback investigations.

**Table 1:** Details of previous AV biofeedback investigations

Investigation author (Year)	Participants	Findings
George [23] (2006)	24 lung cancer patients	• Residual breathing motion within a gating window improved
Venkat [13] (2008)	10 healthy volunteers	• Waveguide breathing guidance produced more regular breathing than bar-model guidance and free breathing
Yang [22] (2012)	Phantom study	• 4D PET image quality improved
An [36] (2013)	Retrospective analysis of George (2006) data	• CTV coverage improved
		• Internal motion variation improved
Kim, [21] Pollock, [37] & Steel [38] (2012–2014)	15 healthy volunteers	• Kim (2012): Breathing regularity of thoracic diaphragm and abdominal wall improved
		• Pollock (2013): Accuracy of kernel density estimation motion prediction improved
		• Steel (2014): Strong correlation between internal and external anatomic motion for both AV biofeedback and free breathing
Lee [24] (2014)	5 healthy volunteers	• Improved 3D MR image quality
		• Reduced gated MRI scan time
Lu [39] (2014)	13 lung & liver cancer patients	• Breathing regularity improved
		• ITV_MIP_ underestimated ITV_10_
Lee [40] (2014)	7 lung cancer patients	• Improved intrafraction lung tumour motion consistency
		• Improved interfraction lung tumour motion consistency

However, none of the studies presented in Table 1 were randomised trials, in addition to this, the findings of a recent literature search yielded that a randomised clinical trial with any breathing guidance intervention has not yet been performed. To fill the gap in the literature, we have designed a multi-institutional, phase II, randomised clinical trial to thoroughly assess the clinical impact of the AV biofeedback breathing guidance system. Based on previous findings, we hypothesise that AV biofeedback will significantly improve breathing regularity and reduce medical imaging errors for lung cancer patients undergoing imaging and treatment procedures during radiotherapy.

This trial has been registered with the Australian New Zealand Clinical Trials Registry (ANZCTR), its trial ID is ACTRN12613001177741.

## Methods/Design

This study aims to assess the clinical impact of AV biofeedback by recruiting 75 lung cancer patients across seven radiation oncology departments. What follows is an outline of the AV biofeedback setup, primary and secondary objectives, participant selection criteria, the study workflow, and statistical considerations for our study design.

### Research Ethics Committee

The protocol for this clinical trial has been reviewed and approved by the Hunter New England Human Research Ethics Committee (HREC). This Human Research Ethics Committee is constituted and operates in accordance with the National Health and Medical Research Council’s ‘National Statement on Ethical Conduct in Human Research (2007)’ (National Statement) and the ‘CPMP/ICH Note of Guidance on Good Clinical Practice’. The Hunter new England HREC has also been accredited by the New South Wales Department of Health as a lead HREC under the single ethical and scientific review. A report on the progress of this clinical trial is required to be submitted annually to the Hunter New England HREC.

### Audiovisual biofeedback system

The AV biofeedback system, as shown in Fig. 1, utilises the Real-time Position Management system (RPM, Varian Medical Systems, Palo Alto, USA) to track the motion of an external marker positioned on the patient’s abdomen. This real-time respiratory-motion is used by the AV biofeedback software to calculate an average cycle of respiration (using a Fourier series fit from 10 obtained respiratory cycles). This average cycle is used as the waveguide (white curve in Fig. 1) which continually moves from right-to-left across the visual display and acts as part of the visual prompt for AV biofeedback. Also on the visual display is a grey marker moving vertically up-and-down corresponding to the anterior-posterior motion of the marker block positioned on the patent’s abdomen. It is the goal for the patient to keep the marker block within inhale-exhale limits (presented as the blue region in Fig. 1) and match the grey marker block over the white waveguide. The audio component of AV biofeedback is classical music playing to the patient; the music fades to silence should the marker block move outside the blue area breathing limits. AV biofeedback has been shown to be compatible in a number of imaging and treatment modalities, [20–22] as well as utilising different types of patient displays [23, 21, 24]. There are two options for patient display in this study: video goggles, or a screen mounted to the couch. Which patient display option is utilised in this study will depend on what is available at each institution.

**Fig. 2:**
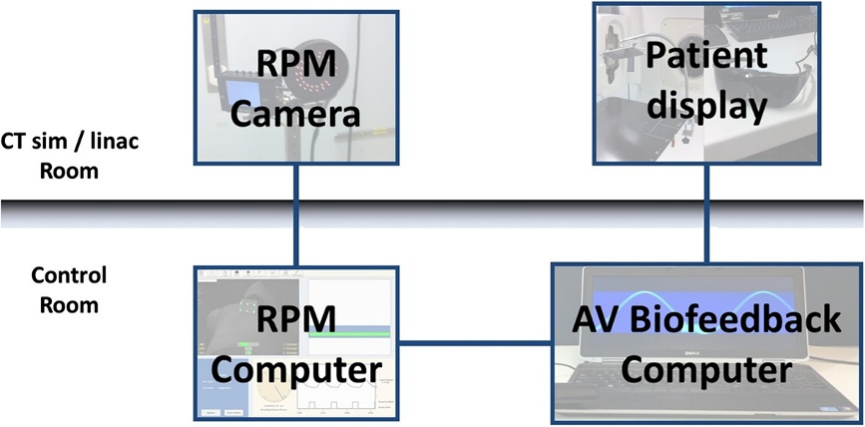
Audiovisual biofeedback study setup schematic

Figure 2 illustrates the schematic of the AV biofeedback study setup, from the RPM camera monitoring patient breathing motion, to the AV biofeedback computer receiving the RPM signal and extending the AV biofeedback guiding interface to the patient display.

### Objectives

This clinical trial will recruit 75 lung cancer patients across 7 radiation oncology departments testing the following objectives:

Primary objective: In a prospective multi-institutional randomised clinical trial we will test the hypothesis that AV biofeedback will significantly improve breathing regularity and reduce medical imaging errors for lung cancer patients undergoing imaging and treatment procedures during radiotherapy.

Secondary objectives will involve patient-specific and department-specific objectives:

Patient-specific objectives are to evaluate the impact of AV biofeedback by:

1. Quantifying the proportion of patients for whom breathing is more regular with AV biofeedback,
2. Quantifying the variability in breathing motion throughout a course of treatment,
3. Quantifying the improvement in image quality with AV biofeedback,
4. Evaluating the patient experience through a perception of care survey,
5. Developing indications and contra-indications for the use of AV biofeedback,
6. Quantifying the differences in image-guided radiotherapy (IGRT) shifts during treatment, and
7. Recording toxicity outcomes for up to 12 months after treatment has been completed.

Department-specific objectives are to evaluate the impact of AV biofeedback on clinical testing by:

1. Quantifying any practice changes (e.g. margin reduction),
2. Quantifying the impact on workflow using the AV biofeedback device through time-motion studies,
3. Evaluating the operator and clinician confidence in the AV biofeedback device’s reliability and clinical efficacy through a technology-impact survey,
4. Quantifying the system robustness through hardware and software fault reporting, and
5. Performing system quality assurance, sharing the results through web-based uploads and provide feedback for QA improvement.
  Our methodological framework will be based on the widely used Technology Acceptance Model (TAM) [25, 26]. The TAM gives qualitative scales for two specific variables, perceived usefulness and perceived ease of use, which are fundamental determinants for user acceptance.

### Study participant selection criteria

This study will recruit patients with cancer of the lung receiving external beam radiation therapy. Patients fitting the eligibility criteria (see below) will be identified and introduced to this study by their treating physicians, who will participate as investigators in this study. The eligibility criteria are as follows:

1. Lung cancer patients
  i. No restrictions to type of external beam radiation therapy being received
  ii. Primary or secondary cancer
2. >18 years old
3. No gender or ethnic restrictions
4. An ECOG score in the range of 0 to 2
5. Able to give written informed consent and willingness to participate and comply with the study
6. No pregnant / lactating woman

### Study workflow

**Fig. 3:**
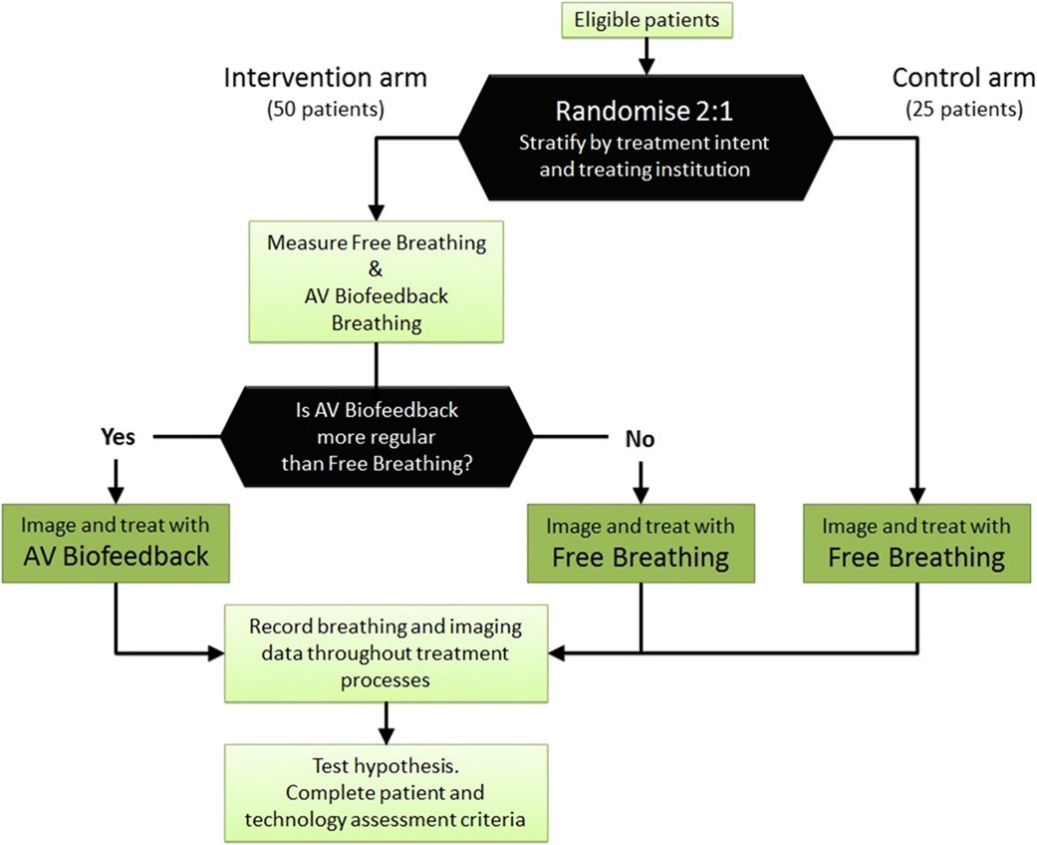
Study flowchart

Once informed consent has been obtained, the patient will be randomised into either the intervention or control arm of the study. For patients randomised into the intervention arm, prior to their planning and treatment they will undergo a breathing decision session during which they will breathe both with and without the guidance of AV biofeedback. Preceding each breathing session will be a training session to familiarise the patient with the AV biofeedback system. After the breathing decision session has been completed, the most reproducible breathing condition (AV biofeedback or free breathing) will be determined in situ by an ‘Analyse Respiratory Session’ function within the AV biofeedback software. It will be the most reproducible breathing condition that will continue to be used throughout the rest of that particular patient’s planning and treatment. The flowchart for this study is shown in Fig. 3.

For all patients, each follow-up visitation they have with their treating physician for the first 12 months after their treatment has finished, their treating physician will complete a toxicity report to satisfy the Secondary Patient-Specific Objective 7: Recording toxicity outcomes for up to 12 months after treatment has been completed by reporting the occurrence and severity of any radiation toxicities.

### Patient randomisation

This trial is stratified, hence, study group random allocation will be determined by minimisation [27, 28]. Patients will be stratified by treating institution and for treatment intent (palliative vs. radical) and minimisation considerably reduces the imbalance of these stratification factors across the control and intervention groups of the study. Patients will be randomised in a 2:1 ratio, 2 out of 3 patients will be randomised into the AV biofeedback (intervention) arm and 1 out of 3 will be randomised into the free breathing (control) arm as illustrated by Fig. 3.

### Sample size and power calculation

The statistical considerations for this study are largely based on a previous study conducted at Virginia Commonwealth University (VCU) on 24 lung cancer patients [23, 29]. Prior to this multi-institutional clinical trial, the VCU study was the largest AV biofeedback investigation, recruiting a total of 26 lung cancer patients, however, 2 patients dropped out due to not being treated with radiotherapy or rapid worsening of disease, and so their data was not collected. In the VCU study 109 breathing sessions were performed comparing AV biofeedback to free breathing, of which, 87 sessions (80 %) demonstrated more regular breathing with AV biofeedback. Framing this is in a more clinical relevant way: irregular breathing motion exacerbates the systematic errors (Σ) arising from motion image artefacts and variations between the planned and treated anatomy, as well as random errors (σ) from day-to-day variations in the treated anatomy [30, 15, 31]. To combine systematic and random errors and estimate the margin contribution due to breathing irregularity we will use the van Herk method [32]: margin = 2.5Σ + 0.7σ, incorporating the respiratory components of systematic and random errors. A clinically significant difference in clinical improvement due to AV biofeedback has been determined to be a margin calculation of less than 5 mm. This magnitude of reduction was elected as clinically significant because this is the same magnitude of displacement attributed to contributing to significant artefacts and errors during radiotherapy procedures as detailed in AAPM Task Group 76 [4]. From this van Herk calculation, in the VCU study there were 14/24 patients with margins <5 mm with AV biofeedback, while only 5/24 for free breathing.

In this proposed study, to get a more accurate indication of the proportion of patients with reduced margins calculated using the van Herk method we have designed an exploratory phase II randomised study examining the potential impact of an AV biofeedback system in regulating breathing in patients receiving radiation therapy for the treatment of lung cancer. Without the AV biofeedback system, it is conservatively estimated that approximately 40 % of patients naturally exhibit regular breathing (margin component below 5 mm). Increasing this proportion to 60 % using the AV biofeedback system would be clinically worthwhile. Based on Simon’s design, [33] a sample size of 50 patients receiving the AV biofeedback system will have at least 80 % power with 95 % confidence to rule out a regular rate of 40 % in favour of a 60 % rate. To minimise patient selection bias and provide an estimate of regular breathing from a contemporary control, the proposed design will be a randomised phase II with a 50 patients receiving the intervention and 25 receiving current standard of care. Patients will be randomised in a 2:1 ratio, with 2/3 of the patients being recruited into the AV biofeedback (intervention) arm and 1/3 in the free breathing (control) arm as illustrated by Fig. 3. 2:1 randomisation is appropriate as within the interventional arm there is a screening procedure where only patients whose breathing is more regular with AV biofeedback use this system for their imaging and treatment procedures. Patients will be stratified by treating institution and for treatment intent (palliative vs. radical) to ensure similar balance in the arms across the sites. As the study is not powered for formal comparisons between the groups, estimates of the proportion of patients which do not experience irregular breathing will provide information as to whether further investigation is warranted.

Assuming a contamination and dropout rate of no more than 10 %, this study will require that 75 + 8 = 83 patients be recruited (the 10 % value was based on the 2/26 patient drop-out rate in the VCU study).

Patients at each institution will be treated per department protocol with no additional constraints on dose, fractionation, immobilisation or image guided procedures. Results will be adjusted for institution (using a fixed effect) to account for differences between institutions.

### Data analysis

The primary objective is to assess the impact of AV biofeedback on breathing regularity and image errors; the section that follows details the metrics to be utilised for the primary objective.

**Fig. 4:**
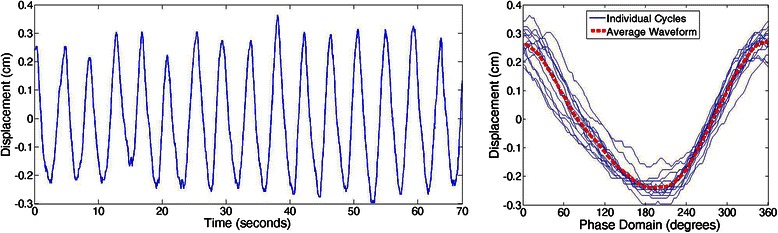
Example of breathing motion trace (left) then separated into individual cycles with the average waveform shown as the red dashed curve (right)

Breathing motion regularity is quantified as the root mean square error (RMSE) in displacement and period [13, 21, 24, 34]. A breathing signal is separated into its individual cycles and an ‘average’ waveform is calculated using a Fourier series fit. Figure 4 illustrates an example breathing trace, its separation into cycles, and its average waveform.

RMSE will be calculated as detailed by Venkat, et al., (2008),[13] but will be outlined here for clarity. For a breathing pattern comprised of *n* individual breathing cycles, where each cycle in the phase domain can be written as *X* = {*x*_1_, *x*_2_, …, *x*_360_} and the average waveform of these cycles can be written as *Y* = {*y*_1_, *y*_2_, …, *y*_360_}, the RMSE in displacement is calculated as:

1 $$ RMSE\  in\  displacement = \frac{{\displaystyle {\Sigma}_{All\  Cycles}}\sqrt{{\displaystyle {\Sigma}_{i=1\dots 360}}\frac{{\left({x}_i\mathit{\hbox{-}}{y}_i\right)}^2}{360}}}{n} $$

The period of each of the $n$ breathing cycles, in seconds, can be written as *P* = {*p*_1_, *p*_2_, …, *p*_*n*_}, with the period of the average waveform expressed as *Period*_*mean*_, the RMSE in period is calculated as:

2 $$ RMSE\  in\  period = \sqrt{\frac{{\displaystyle {\varSigma}_{i=1\dots n}}{\left({p}_i\mathit{\hbox{-}} Perio{d}_{mean}\right)}^2}{n}} $$

The impact of AV biofeedback on 4D-CT image quality will utilise an automated method of image artefact identification developed by Cui, et al., (2012), [35] but will be outlined here for clarity. The method is based on the similarity between edge slices at adjacent couch positions *A* and *B*; the edge similarity between slice *A* and slice *B* is expressed by the normalised correlation coefficient (NCC). Deviations from standard NCC, representing normal anatomical changes between edge slices, signify the presence of an image artefact. Cui, et al., (2012) reported good agreement of their method with the assessment of two observers.

## Discussion

This clinical trial marks an important milestone in breathing guidance studies as it will be the first randomised, controlled trial providing the most comprehensive evaluation of the clinical impact of breathing guidance on cancer radiation therapy to date. Based on the structure of previous investigations, and taking into consideration the increase in scope of this study, the authors have designed a multi-institutional, randomised, phase II, stratified clinical trial to test the hypothesis that audiovisual biofeedback breathing guidance will significantly improve breathing regularity and reduce medical imaging errors for lung cancer patients undergoing imaging and treatment procedures during radiotherapy. While patients will be stratified by treating institution and for treatment intent, the study is not powered for formal comparisons between the these stratified groups; estimates from the current proposed study of the proportion of patients which do not experience irregular breathing will provide information as to whether further investigation is warranted. Further to this, objectives such as determining the indications and contra-indications for the use of audiovisual biofeedback, evaluation of patient experience, radiation toxicity occurrence and severity, and clinician confidence will shed light on the design of future phase III clinical trials.

## Abbreviations

AV biofeedback: Audiovisual biofeedback
HREC: Human Research Ethics Committee
PET: Positron Emission Tomography
MRI: Magnetic resonance imaging
ANZCTR: Australian New Zealand Clinical Trials Registry
RPM: Real-time position management
IGRT: Image-guided radiotherapy
QA: Quality assurance
TAM: Technology acceptance model
ECOG score: Eastern cooperative oncology group score
VCU: Virginia Commonwealth University
